# Nasal or throat sampling is adequate for the detection of the human respiratory syncytial virus in children with acute respiratory infections

**DOI:** 10.1002/jmv.25496

**Published:** 2019-05-26

**Authors:** Van Hoan Nguyen, Fiona M Russell, David AB Dance, Keoudomphone Vilivong, Souphatsone Phommachan, Chanthaphone Syladeth, Jana Lai, Ruth Lim, Melinda Morpeth, Mayfong Mayxay, Paul N Newton, Xavier De Lamballerie, Audrey Dubot‐Pérès

**Affiliations:** ^1^ Unité des Virus Émergents (UVE: Aix‐Marseille Univ ‐ IRD 190 ‐ Inserm 1207 ‐ IHU Méditerranée Infection) Marseille France; ^2^ Department of Paediatrics The University of Melbourne Melbourne Australia; ^3^ Pneumococcal Research Group, Murdoch Children's Research Institute The Royal Children's Hospital Melbourne Australia; ^4^ Microbiology Laboratory Lao‐Oxford‐Mahosot Hospital‐Wellcome Trust Research Unit (LOMWRU) Vientiane Capital Lao PDR; ^5^ Nuffield Department of Clinical Medicine, Centre for Tropical Medicine and Global Health University of Oxford Oxford United Kingdom; ^6^ Faculty of Infectious and Tropical Diseases London School of Hygiene and Tropical Medicine London United Kingdom; ^7^ Institute of Research and Education Development University of Health Sciences Vientiane Lao PDR

**Keywords:** detection rate, human respiratory syncytial virus, Laos, nasal swab, nasopharyngeal swab, throat swab

## Abstract

Human respiratory syncytial virus (HRSV) is one of the most important causes of acute respiratory infections (ARI) in young children. HRSV diagnosis is based on the detection of the virus in respiratory specimens. Nasopharyngeal swabbing is considered the preferred method of sampling, although there is limited evidence of the superiority of nasopharyngeal swabs (NPS) over the less invasive nasal (NS) and throat (TS) swabs for virus detection by real‐time reverse transcription quantitative polymerase chain reaction (RT‐qPCR). In the current study, we compared the three swabbing methods for the detection of HRSV by RT‐qPCR in children hospitalized with ARI at Mahosot Hospital, Vientiane, Laos. In 2014, NS, NPS, and TS were collected from 288 children. All three samples were tested for HRSV by RT‐qPCR; 141 patients were found positive for at least one sample. Almost perfect agreements (*κ* > 0.8) between the swabs, compared two by two, were observed. Detection rates for the three swabs (between 93% and 95%) were not significantly different, regardless of the clinical presentation. Our findings suggest that the uncomfortable and technically more demanding NPS method is not mandatory for HRSV detection by RT‐qPCR.

## 1 INTRODUCTION

Human respiratory syncytial virus (HRSV) is a common respiratory pathogen in children under the age of 5 years. In 2015, there were estimated to be 33.1 million new episodes of HRSV‐associated acute lower respiratory infections worldwide, of which 3.2 million were hospitalized and 59 600 patients died.^1^ HRSV diagnosis is based on the detection of the virus in respiratory specimens using cell culture, immunofluorescence, immunoenzymatic, or molecular assays. During the past decade, polymerase chain reaction (PCR), a fast and accurate detection tool, has been widely used in the diagnosis and is often chosen over conventional methods for the detection of respiratory pathogens.^2^

Collection of nasopharyngeal swabs (NPS) is considered the preferred sampling method for the detection of respiratory viruses,^3^ although it requires experienced staff and can be uncomfortable, especially for young children. There is limited evidence of the superiority of NPS over the less invasive nasal (NS) and throat (TS) swabs for virus detection by real‐time PCR, with only a few studies evaluating HRSV detection in children^4^, ^5^, ^6^, ^7^ and two including NS.^6^, ^7^ We are not aware of studies that have compared all three sampling methods.

In 2014, we conducted a study on children (<5‐year‐old) hospitalized at Mahosot Hospital, Vientiane, Laos, with an acute respiratory infection (ARI).^8^ Three different samples (NS, TS, and NPS) were collected from a large proportion of these patients. Since HRSV was one of the most common pathogens detected, this gave us the opportunity to compare the performance of these three sampling techniques for the detection of HRSV by real‐time reverse transcription quantitative polymerase chain reaction (RT‐qPCR).

## 2 MATERIALS AND METHODS

### 2.1 Specimen collection

From December 2013 to December 2014, 383 children younger than 5 years of age, with a clinical presentation of ARI were enrolled, as previously described.^8^ At inclusion, samples were collected at the same time in the following sequence: TS, NS, then NPS. They were available for 288 (75.2%) patients who were included in this study. NS and TS were placed separately in 1 mL viral transport medium (Sigma Virocult [MWE]), Corsham, England vials. NPS was placed in 1 mL of skim‐milk tryptone glucose glycerol medium (STGG), to allow subsequent bacterial and viral investigations from the same sample.^9^ Virocult vials and STGG were transported to the laboratory within 2 hours in a cool box. Swabs were squeezed, and the media were aliquoted and stored at −80°C before performing the laboratory assays.

### 2.2 Testing for HRSV

Nucleic acids were extracted from 100 µL of each swab medium using the Cador Pathogen 96 QIAcube HT kit (Qiagen, Hilden, Germany) following the manufacturer's instructions, with an elution volume of 90 µL. RT‐qPCR for HRSVA/B detection was performed using specific primers and probes as described by Bonroy et al^10^ Testing was performed following the manufacturer's instructions, using the Express One‐Step Superscript qRT‐PCR Universal Kit (Thermo Fisher Scientific, Waltham, MA), 5 µL of RNA, 500 nM of each primer and 200 nM of probe in a final reaction volume of 20 µL. The limit of detection of this HRSV RT‐qPCR assay is 9.5 copies/µL, estimated using triplicates of 1/5 serial dilutions of quantified synthetic RNA. Amplification and detection were performed with the QuantStudio 12K Flex Real‐time PCR system instrument (Applied Biosystems, Foster City, CA). The thermal cycling was: 15 minutes at 50°C, 2 minutes at 95°C, followed by 40 cycles of 15 seconds at 95°C and 30 seconds at 60°C. Negative and positive controls were added to each run. Samples with *C*q value less than 35 were considered as positive for HRSV.

### 2.3 HRSV quantification

RNA (4.93·10^6^ copies/μL, quantified by RT‐qPCR using a quantified synthetic RNA prepared as previously described^11^), was extracted from an HRSVA strain (UVE/HRSV‐A/2011/FR/3506, reference 001V‐02477 provided by the EVA collection https://www.european‐virus‐archive.com/) and used as a positive control. Ten‐fold serial dilutions of the RNA (1, 10^−1^, 10^−2^, 10^−3^, and 10^−4^) were prepared, aliquoted and stored at −80°C to be used as standards. One aliquot of each standard was added to each RT‐qPCR run, then the standard curve was drawn for the quantification of each tested sample.

### 2.4 Statistical analysis

Patients were classified as HRSV‐positive if at least one of the three swabs were found positive by RT‐qPCR. The detection rate was calculated for a given swab as the percentage of HRSV patients detected. Agreements of the HRSV RT‐qPCR results between the swabs, compared two by two, were assessed by calculating the *κ* coefficient. The *κ* results were interpreted as follows: values 0 or lower as indicating no agreement, and 0.01‐0.20 as none to slight, 0.21‐0.40 as fair, 0.41‐0.60 as moderate, 0.61‐0.80 as substantial, and 0.81‐1.00 as almost perfect agreement.^12^ Calculation of 95% confidence intervals (CI) was performed using Statistical Package for the Social Sciences version 23.0 for Windows (SPSS Inc, Chicago, IL).

### 2.5 Ethics statement

The study was conducted according to the protocol approved by the National Ethics Committee for Health Research, Ministry of Health, Lao PDR, and the Oxford Tropical Research Ethics Committee. Signed, informed consent was obtained from the parents of each child included in this study.

## 3 RESULTS

The median (interquartile range [IQR]) age of the 288 patients included was 14 months (7‐23 months); 165 (57.3%) were male. The characteristics of the patients are presented in supplemental data (Table S1).

**Figure 1:**
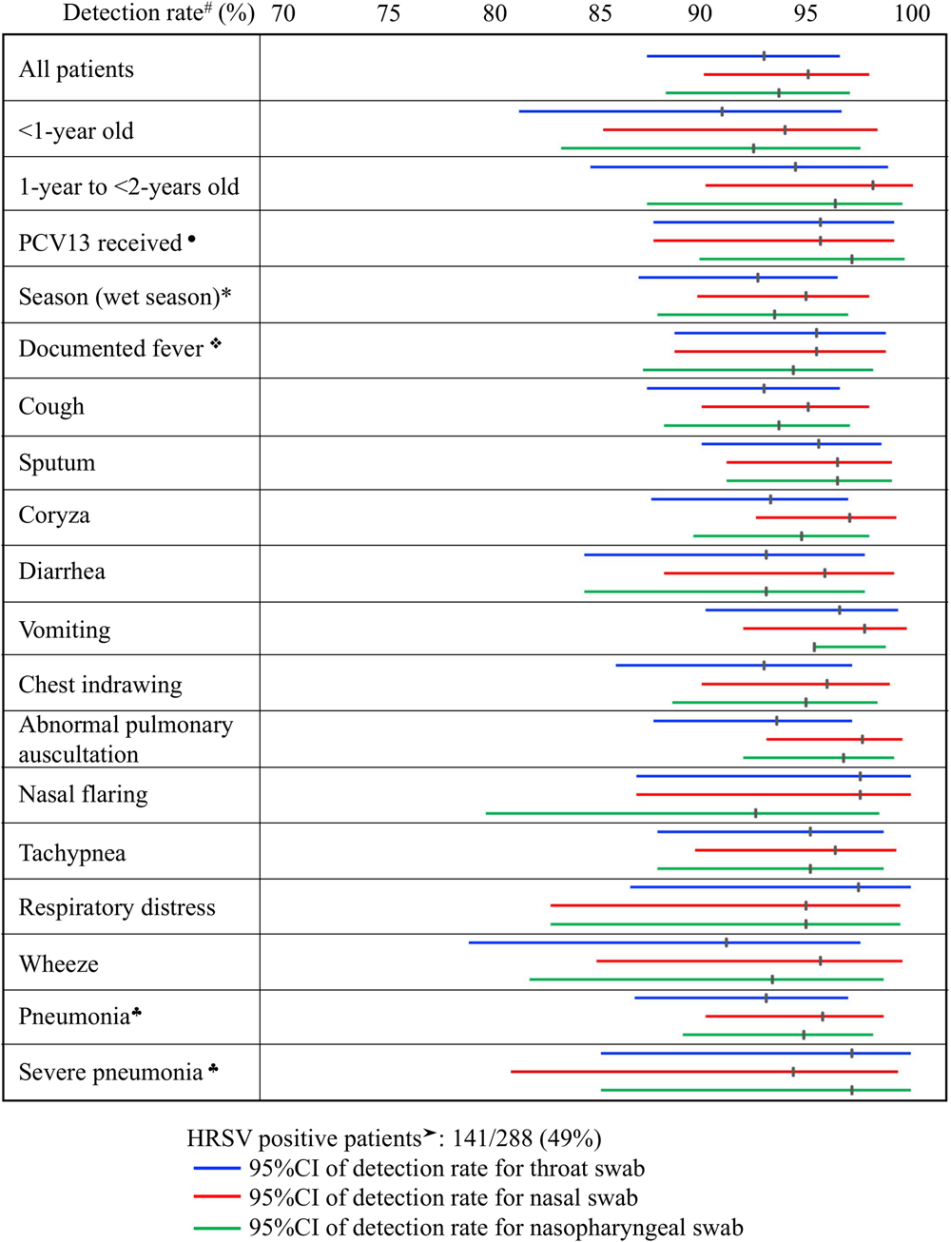
Detection rate of the three swabs tested for the detection of HRSV by RT‐qPCR according to patient characteristics. Only the characteristics which were observed in more than 30 HRSV‐positive patients are displayed. ^#^Detection rate of each swab for the detection of HRSV by RT‐qPCR calculated over the total number of positive patients (positive in at least one of the three swabs tested). ●“PCV13 received” if they had received at least two doses of vaccine for children less than 1‐year‐old or at least one dose of vaccine for children between 1‐ to 2‐year old. *wet season: from May to October. ■Low birth weight: defined by the World Health Organisation (WHO) as weight at birth less than 2500 g. ❖Fever: defined as body temperature 38°C or higher per axilla. ➤HRSV‐positive patients = positive for HRSV by RT‐qPCR for at least one of the three swabs tested. ♣Pneumonia and severe pneumonia were defined according to WHO criteria: children who presented with cough or difficulty breathing and had fast breathing (aged 2‐11 months: ≥50 breaths/minute, aged 1‐4 years: ≥40 breaths/min) or chest indrawing, were classified as having pneumonia; children who presented with cough or difficulty breathing and had at least one of the following criteria were classified as severe pneumonia: oxygen saturation 90% or lesser , while breathing room air, or central cyanosis; severe respiratory distress; signs of pneumonia with a general danger sign (inability to breastfeed or drink, lethargy or reduced level of consciousness, convulsions, vomiting). Children younger than 2‐month old who presented with cough or difficulty breathing and fast breathing (≥60 breaths/min) were classified as severe pneumonia. HRSV, human respiratory syncytial virus; RT‐qPCR, reverse transcription quantitative polymerase chain reaction

One hundred and forty‐one (49.0%) patients were found positive for HRSV from at least one sample type. TS was positive in 131 (45.5%) patients, NS in 134 (46.5%), and NPS in 132 (45.8%). Almost perfect agreements (κ > 0.80) between the three swabs, compared two by two, for HRSV RT‐qPCR results were observed (Table 1). No significant difference was observed between the detection rates calculated for each specimen type: 92.9% (95% CI: 87.3‐96.5) for TS, 95.0% (95% CI: 90.0‐98.0) for NS, and 93.6% (95% CI: 88.2‐97.0) for NPS (Figure 1 and Tables 1 and S1).

**Table 1:** Detection rates of the three swabs tested for the detection of HRSV by RT‐qPCR

Characteristics	All ARI patients, n (%)	HRSV‐positive,^a^ n (%)	TS		NS		NPS		*κ* ^b^ (95% CI)		
			HRSV positive, n (%)	Det rate,^c^ % (95% CI)	HRSV positive, n (%)	Det rate,^c^ % (95% CI)	HRSV positive, n (%)	Det rate,^c^ % (95% CI)	TS‐NS	TS‐NPS	NS‐NPS
Number of patients	288	141	131	92.9 (87.3‐96.5)	134	95.0 (90.0‐97.9)	132	93.6 (88.2‐97.0)	0.89 (0.87‐0.91)	0.89 (0.86‐0.91)	0.95 (0.94‐0.96)
Age, median (IQR), mo	7 (14‐23)	6 (13‐20)	6 (13‐20)		6 (13‐20)	–	6 (13‐20)	–	–	–	–
Age groups, y											
<1	117 (40.6)	66 (46.8)	60 (45.8)	90.9 (81.2‐96.6)	62 (46.3)	93.9 (85.2‐98.3)	61 (46.2)	92.4 (83.2‐97.5)	0.82 (0.76‐0.87)	0.85 (0.78‐0.89)	0.95 (0.92‐0.96)
1 to <2	100 (34.7)	54 (38.3)	51 (38.9)	94.4 (84.6‐98.8)	53 (39.6)	98.1 (90.1‐100)	52 (39.4)	96.3 (87.3‐99.5)	0.96 (0.94‐0.97)	0.94 (0.91‐0.95)	0.94 (0.91‐0.95)
2 to <5	71 (24.7)	21 (14.9)	20 (15.3)	95.2 (76.2‐99.9)	19 (14.2)	90.5 (69.6‐98.8)	19 (14.4)	90.5 (69.6‐98.8)	0.89 (0.83‐0.93)	0.89 (0.83‐0.93)	1.00
Gender (male)	165 (57.3)	83 (58.9)	77 (58.9)	92.8 (84.9‐97.3)	78 (58.2)	94.0 (86.5‐98.2)	77 (58.3)	92.8 (84.9‐97.3)	0.86 (0.82‐0.90)	0.87 (0.83‐0.90)	0.96 (0.95‐0.97)

Abbreviations: ARI, acute respiratory infections; CI, confidence interval; HRSV, human respiratory syncytial virus; IQR, iInterquartile range; NPS: nasopharyngeal swab; NS: nasal swab; RT‐qPCR, reverse transcription quantitative polymerase chain reaction; TS: throat swab.

^a^ HRSV positive patients = positive for HRSV by RT‐qPCR for at least one of the three swabs tested**.**

^b^ κ Coefficient measures the agreement of the HRSV RT‐qPCR results between the swabs compared two by two.

^c^ Det rate = Detection rate of each swab for the detection of HRSV by RT‐qPCR calculated over the number of patients positive in any of the three swabs.

Detection rates of the three sampling techniques were analyzed according to demographic and clinical patient characteristics along with their 95% CI (Figure 1 and Table S1). No significant difference was observed between the three different swabs for any of the characteristics analyzed, even after stratification by age, gender, and clinical presentation (including with or without coryza). However, only seven HRSV positive patients presented with no coryza.

**Figure 2:**
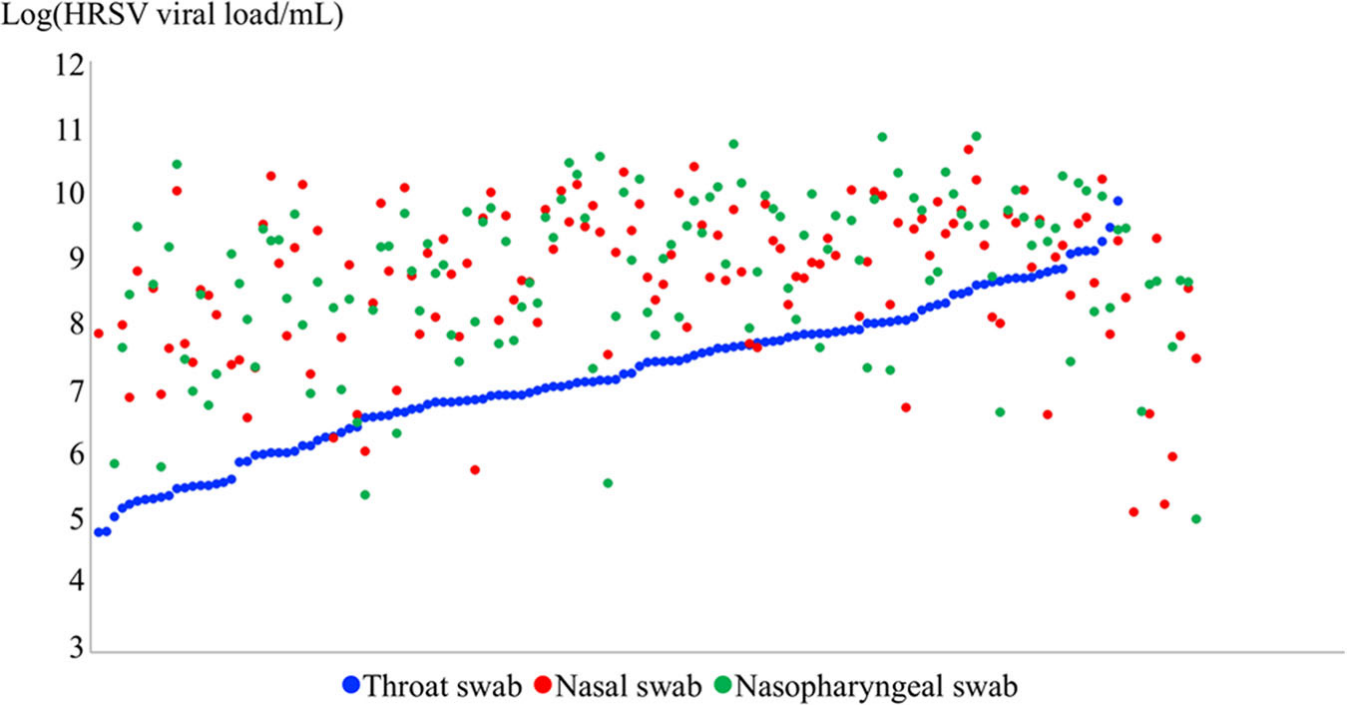
Comparison of human respiratory syncytial virus (HRSV) viral loads detected in throat, nasal, and nasopharyngeal swabs for all patients tested. Patients are distributed along the *X*‐axis, sorted by increasing HRSV viral load detected in throat swab

The median (IQR) of HRSV viral load detected was 1.3 × 10^7^ copies/mL (2.3 × 10^6^‐9.3 × 10^7^) in TS, 6.9 × 10^8^ copies/mL (8.8 × 10^7^‐3.2 × 10^9^) in NS, and 8.8 × 10^8^ copies/mL (1.1 × 10^8^‐4.3 × 10^9^) in NPS. HRSV viral load was on average significantly lower in TS than in NS and in NPS (*P* < .001, *t* test). When patients were sorted by increasing TS viral load, we observed that the viral load in TS was lower than in NS and NPS for most patients, 90% and 91%, respectively (Figure 2).

## 4 DISCUSSION

Although it is often considered a preferred method for the detection of respiratory pathogens, our study showed that NPS was not significantly better than NS or TS for the detection of HRSV by RT‐qPCR in Lao children, with almost perfect agreements (*κ* > 0.80) between the swabs observed. The detection rates for the three swabs (between 93% and 95%) were not significantly different. In addition, the HRSV viral load detected in NS was not significantly different to that in NPS (*P* > .05, *t* test), but significantly higher than that in TS (*P* < .001, *t* test).

Our findings are in accordance with previous publications. Grijalva et al^6^ found good agreement between NS and NPS for the detection of HRSV. However, NPS was not systematically investigated for all patients and the study included only 36 HRSV patients. Dawood et al^7^ observed high detection rates for both NS and TS (98% and 93%, respectively) for the detection of 343 HRSV patients from 703 hospitalized children. However, they did not investigate NPS.

We also investigated whether the choice of the sampling method should be based on particular patient characteristics, such as young age, specific respiratory symptoms, or signs of severity. For this, the detection rates of the three swabs were calculated and compared within different groups of patients sharing the same characteristics. No significant difference was observed between the three different swabs for any of the patient groups tested. However, most of the patients included in this study had coryza (90%), so the values of the detection rate for the three swabs could not be established with accuracy for the seven HRSV patients with no coryza.

Accurate diagnosis is closely linked to the quality of the sample collection, which could be impacted, amongst other things, by the practicability of the sampling method and its acceptance by the patient and their family. Our study provides evidence that a simple and painless NS sampling can be used with a high degree of accuracy for the detection of HRSV by RT‐qPCR in children hospitalized for ARI presenting with coryza. This is of particular importance, especially in young children for whom NPS sampling is unpleasant and can be challenging when performed by less experienced staff. When available, simple and painless methods should be prioritized after appropriate validation. However, our study was limited to the assessment of HRSV detection in children less than 5 years of age, therefore extrapolation of our findings to other age groups and/or other respiratory viruses would require additional investigations.

We conclude that performing NS sampling is appropriate for the molecular detection of HRSV in children under the age of 5 years. Further investigations are needed for systematic comparison of all swabbing methods in different clinical contexts and for an extended panel of respiratory pathogens.

## CONFLICT OF INTERESTS

The authors declare that there are no conflict of interests.

## Supporting information

Supporting information

Supporting information
