# Supplementary material for: Nasal or throat sampling is adequate for the detection of the human respiratory syncytial virus in children with acute respiratory infections
Source: J Med Virol. 2019 May 26;91(9):1602–7. doi: 10.1002/jmv.25496 (PMC6772119; doi:10.1002/jmv.25496)
Supplement: Supplementary file 1 — Supporting information [file JMV-91-1602-s001.doc]

**Supplemental data: Characteristics of patients included and sensitivities of the three swabs tested for the detection of HRSV by RT-qPCR.**

| **Characteristics** | **All ARI patients, n (%)** | **HRSV positive*, n (%)** | **Throat swab** | | **Nasal swab** | | **Nasopharyngeal swab** | | **Kappa**^⁋^ **(95%CI)** | | |
| --- | --- | --- | --- | --- | --- | --- | --- | --- | --- | --- | --- |
|  |  |  | **HRSV positive, n (%)** | **Sensitivity**^#^**,% (95%CI)** | **HRSV positive, n (%)** | **Sensitivity**^#^**, %(95%CI)** | **HRSV positive, n (%)** | **Sensitivity**^#^**, %(95%CI)** | **TS-NS paired** | **TS-NPS paired** | **NS-NPS paired** |
| **Number of patients** | 288 | 141 | 131 | 92.9 (87.3-96.5) | 134 | 95.0 (90.0-97.9) | 132 | 93.6 (88.2-97.0) | 0.89 (0.87-0.91) | 0.89 (0.86-0.91) | 0.95 (0.94-0.96) |
| **Age (months), median (IQR)** | 7 (14-23) | 6 (13-20) | 6 (13-20) |  | 6 (13-20) |  | 6 (13-20) |  |  |  |  |
| **Age groups** |  |  |  |  |  |  |  |  |  |  |  |
| < 1-year old | 117 (40,6) | 66 (46,8) | 60 (45,8) | 90.9 (81,2-96.6) | 62 (46,3) | 93,9 (85,2-98,3) | 61 (46,2) | 92,4 (83,2-97,5) | 0.82 (0.76-0.87) | 0.85 (0.78-0.89) | 0.95 (0.92-0.96) |
| 1 to < 2-years old | 100 (34.7) | 54 (38.3) | 51 (38.9) | 94.4 (84.6-98.8) | 53 (39.6) | 98.1 (90.1-100) | 52 (39.4) | 96.3 (87.3-99.5) | 0.96 (0.94-0.97) | 0.94 (0.91-0.95) | 0.94 (0.91-0.95) |
| 2 to < 5-years old | 71 (24.7) | 21 (14.9) | 20 (15.3) | 95.2 (76.2-99.9) | 19 (14.2) | 90.5 (69.6-98.8) | 19 (14.4) | 90.5 (69.6-98.8) | 0.89 (0.83-0.93) | 0.89 (0.83-0.93) | 1.00 |
| **Gender (male)** | 165 (57.3) | 83 (58.9) | 77 (58.9) | 92.8 (84.9-97.3) | 78 (58.2) | 94.0 (86.5-98.2) | 77 (58.3) | 92.8 (84.9-97.3) | 0.86 (0.82-0.90) | 0.87 (0.83-0.90) | 0.96 (0.95-0.97) |
| **Low birth weight at birth**^■^ | 36 (12.5) | 20 (14.2) | 19 (14.5) | 95.0 (75.1-99.9) | 17 (12.7) | 85.0 (62.1-96.8) | 16 (12.1) | 80.0 (56.3-94.3) | 0.78 (0.61-0.88) | 0.73 (0.53-0.85) | 0.94 (0.89-0.97) |
| **PVC 13 received**^●^ | 125 (43.4) | 68 (48.3) | 65 (49.6) | 95.6 (87.6-99.1) | 65 (48.5) | 95.6 (87.6-99.1) | 66 (50.0) | 97.1 (89.8-99.6) | 0.90 (0.86-0.93) | 0.92 (0.88-0.94) | 0.98 (0.97-0.99) |
| **Season (wet season)*** | 222 (77.1) | 136 (96.5) | 126 (96.2) | 92.6 (86.9-96.4) | 129 (96.3) | 94.9 (89.7-97.9) | 127 (96.2) | 93.4 (87.8-96.9) | 0.86 (0.82-0.89) | 0.86 (0.82-0.89) | 0.94 (0.92-0.95) |
| **Clinical presentation** |  |  |  |  |  |  |  |  |  |  |  |
| Documented fever^❖^ | 188 (65.3) | 87 (61.7) | 83 (63.4) | 95.4 (88.6-98.7) | 83 (61.9) | 95.4 (88.6-98.7) | 82 (62.1) | 94.3 (87.1-98.1) | 0.93 (0.91-0.95) | 0.92 (0.90-0.94) | 0.96 (0.95-0.97) |
| Cough | 281 (97.6) | 140 (99.3) | 130 (99.2) | 92.9 (87.3-96.5) | 133 (99.3) | 95.0 (89.9-97.9) | 131 (99.2) | 93.6 (88.1-97.0) | 0.89 (0.86-0.91) | 0.89 (0.86-0.91) | 0.95 (0.94-0.96) |
| Difficulty breathing | 203(70.5) | 116 (82.3) | 107 (81.6) | 92.2 (85.7-96.3) | 112 (83.6) | 96.6 (91.4-99.0) | 111 (84.1) | 95.7 (90.2-98.5) | 0.89 (0.86-0.91) | 0.88 (0.84-0.90) | 0.95 (0.93-0.96) |
| Sputum | 195 (67.7) | 112 (79.4) | 107 (81.7) | 95.5 (89.9-98.5) | 108 (80.6) | 96.4 (91.1-99.0) | 108 (81.8) | 96.4 (91.1-99.0) | 0.92 (0.90-0.94) | 0.90 (0.87-0.92) | 0.95 (0.94-0.96) |
| Coryza | 259 (89.9) | 133 (94.3) | 124 (94.7) | 93.2 (87.5-96.9) | 129 (96.3) | 97.0 (92.5-99.2) | 126 (95.5) | 94.7 (89.5-97.9) | 0.90 (0.87-0.92) | 0.90 (0.88-0.92) | 0.96 (0.95-0.97) |
| No coryza | 28 (9.7) | 7 (5.0) | 6 (4.6) | 85.7 (42.1-99.6) | 4 (3.0) | 57.1 (18.4-90.1) | 5 (3.8) | 71.4 (29.0-96.3) | 0.77 (0.56-0.88) | 0.66 (0.39-0.82) | 0.87 (0.74-0.93) |
| Diarrhea | 128 (44.4) | 71 (50.4) | 66 (50.4) | 93.0 (84.3-97.7) | 68 (50.7) | 95.8 (88.1-99.1) | 66 (50.0) | 93.0 (84.3-97.7) | 0.87 (0.82-0.91) | 0.84 (0.78-0.88) | 0.96  (0.95-0.97) |
| Nausea | 10 (3.5) | 7 (5) | 6 (4.6) | 85.7 (42.1-99.6) | 6 (4.5) | 85.7 (42.1-99.6) | 6 (4.5) | 85.7 (42.1-99.6) | 0.58 (0.29-0.87) | 0.58 (0.29-0.87) | 1.00 |
| Vomiting | 158 (54.9) | 86 (61) | 83 (63.4) | 96.5 (90.1-99.3) | 84 (62.7) | 97.7 (91.9-99.7) | 82 (62.1) | 95.3 (88.5-98.7) | 0.93 (0.91-0.95) | 0.91 (0.88-0.93) | 0.97  (0.96-0.98) |
| Rigors | 21 (7.3) | 6 (4.3) | 6 (4.6) | 100 (54.1-100) | 6 (4.5) | 100 (54.1-100) | 6 (4.5) | 100 (54.1-100) | 1.00 | 1.00 | 1.00 |
| Convulsions | 27 (9.4) | 5 (3.5) | 4 (3.1) | 80.0 (28.4-99.5) | 5 (3.7) | 100 (47.8-100) | 5 (3.4) | 100 (47.8-100) | 0.87 (0.73-0.93) | 0.87 (0.73-0.93) | 1.00 |
| **Physical examination** |  |  |  |  |  |  |  |  |  |  |  |
| Chest indrawing | 158 (54.9) | 98 (69.5) | 91 (69.5) | 92.9 (85.8-97.1) | 94 (70.1) | 95.9 (89.9-98.9) | 93 (70.5) | 94.9 (88.5-98.3) | 0.88 (0.84-0.91) | 0.87 (0.82-0.90) | 0.93 (0.91-0.95) |
| Abnormal pulmonary auscultation | 199 (69.1) | 123 (87.2) | 115 (87.8) | 93.5 (87.6-97.1) | 120 (89.6) | 97.6 (93.0-99.5) | 119 (90.2) | 96.7 (91.9-99.1) | 0.90 (0.88-0.92) | 0.89 (0.86-0.92) | 0.94 (0.93-0.96) |
| Lymphadenopathy | 10 (3.5) | 1 (0.7) | 1 (0.8) | 100 (2.5-100) | 1 (0.7) | 100 (2.5-100) | 1 (0.8) | 100 (2.5-100) | 1.00 | 1.00 | 1.00 |
| Grunting | 13 (4.5) | 5 (3.5) | 4 (3.1) | 80.0 (28.4-99.5) | 5 (3.7) | 100 (47.8-100) | 5 (3.8) | 100 (47.8-100) | 0.84 (0.56-0.94) | 0.84 (0.56-0.94) | 1.00 |
| Nasal flaring | 65 (22.6) | 40 (28.4) | 39 (29.8) | 97.5 (86.8-99.9) | 39 (29.1) | 97.5 (86.8-99.9) | 37 (28.0) | 92.5 (79.6-98.4) | 0.93 (0.89-0.96) | 0.93 (0.90-0.96) | 0.93 (0.90-0.96) |
| Tachypnea | 136 (47.2) | 81 (59.6) | 77 (58.8) | 95.1 (87.8-98.6) | 78 (58.2) | 96.3 (89.6-99.2) | 77 (58.3) | 95.1 (87.8-98.6) | 0.92 (0.89-0.94) | 0.91 (0.87-0.93) | 0.95 (0.93-0.96) |
| Oxygen saturation <90% | 34 (11.8) | 15 (57.4) | 15 (11.5) | 100 (78.2-100) | 14 (10.4) | 93.3 (68.1-99.8) | 14 (10.6) | 93.3 (68.1-99.8) | 0.94 (0.88-0.97) | 0.94 (0.88-0.97) | 1.00 |
| Rashes | 17 (5.9) | 5 (3.5) | 5 (3.8) | 100 (47.8-100) | 4 (3.0) | 80.0 (28.4-99.5) | 4 (3.0) | 80.0 (28.4-99.5) | 0.85 (0.65-0.94) | 0.85 (0.65-0.94) | 1.00 |
| Respiratory distress | 72 (25.0) | 39 (27.7) | 38 (29.0) | 97.4 (86.5-99.9) | 37 (27.6) | 94.9 (82.7-99.4) | 37 (28.0) | 94.9 (82.7-99.4) | 0.97 (0.95-0.98) | 0.91 (0.87-0.94) | 0.94 (0.91-0.96) |
| Cyanosis | 28 (9.7) | 15 (10.6) | 15 (11.5) | 100 (78.2-100) | 14 (10.4) | 93.3 (68.1-99.8) | 14 (10.6) | 93.3 (68.1-99.8) | 0.93 (0.85-0.96) | 0.93 (0.85-0.96) | 1.00 |
| Wheeze | 80 (27.8) | 45 (31.9) | 41 (31.3) | 91.1 (78.8-97.5) | 43 (32.1) | 95.6 (84.9-99.5) | 42 (31.8) | 93.3 (81.7-98.6) | 0.85 (0.77-0.90) | 0.85 (0.81-0.91) | 0.92 (0.88-0.95) |
| Stridor | 11 (3.8) | 2 (1.4) | 2 (1.5) | 100 (15.8-100) | 1 (0.7) | 50.0 (1.3-98.7) | 1 (0.8) | 50.0 (1.3-98.7) | 0.64 (0.10-0.88) | 0.64 (0.10-0.88) | 1.00 |
| Inability to drink | 25 (8.7) | 13 (9.2) | 12 (9.2) | 92.3  (63.9-99.8) | 11 (8.2) | 84.6 (54.6-98.1) | 12 (9.1) | 92.3 (64.0-99.8) | 0.76 (0.53-0.88) | 0.84 (0.67-0.92) | 0.92 (0.83-0.96) |
| Prostration or lethargy | 16 (5.6) | 6 (4.3) | 5 (3.8) | 83.3  (35.9-99.6) | 6 (4.5) | 100 (54.1-100) | 6 (4.5) | 100 (54.1-100) | 0.87 (0.66-0.95) | 0.87 (0.66-0.95) | 1.00 |
| **Sub-groups** |  |  |  |  |  |  |  |  |  |  |  |
| Pneumonia^^ | 203 (70.5) | 115 (81.6) | 107 (81.7) | 93.0 (86.7-96.9) | 110 (82.1) | 95.7 (90.1-98.6) | 109 (82.6) | 94.8 (89.0-98.1) | 0.89 (0.85-0.91) | 0.88 (0.84-0.90) | 0.95 (0.93-0.96) |
| Severe pneumonia^^ | 89 (30.9) | 35 (24.8) | 34 (26.0) | 97.1 (85.1-99.9) | 33 (24.6) | 94.3 (80.8-99.3) | 34 (25.8) | 97.1 (85.1-99.9) | 0.92 (0.89-0.95) | 0.95 (0.92-0.96) | 0.97 (0.96-0.98) |

*HRSV positive patients=positive for HRSV by RT-qPCR for at least one of the three swabs tested**.** ^#^Sensitivity of each swab for the detection of HRSV by RT-qPCR calculated over the number of patients positive in any of the three swabs. TS: throat swab, NS: nasal swab, NPS: nasopharyngeal swab. ^⁋^Kappa coefficient measures agreement of the HRSV RT-qPCR results between the swabs, compared two by two. ^●^‘PCV13 received’ if they had received at least two doses of vaccine for children less than 1 year old or at least one dose of vaccine for children between 1 to 2 years old. *wet season: from May to October. ^■^Low birth weight at birth: defined by World Health Organization as weight at birth less than 2,500 g. ^❖^Fever: defined as body temperature ≥38°C per axilla. ^^Pneumonia and severe pneumonia were defined according to WHO criteria (14). Children who presented with cough or difficulty breathing and had fast breathing (aged 2–11 months: ≥50 breaths/minute, aged 1–4 years: ≥40 breaths/minute) or chest indrawing, were classified as having pneumonia. Children who presented with cough or difficulty breathing and had at least one of the following criteria were classified as severe pneumonia: oxygen saturation <90%, while breathing room air, or central cyanosis; severe respiratory distress; signs of pneumonia with a general danger sign (inability to breastfeed or drink, lethargy or reduced level of consciousness, convulsions, vomiting). Children <2 months old who presented with cough or difficulty breathing and fast breathing (≥60 breaths/min) were classified as severe pneumonia.
