# Supplementary material for: Nasal or throat sampling is adequate for the detection of the human respiratory syncytial virus in children with acute respiratory infections
Source: J Med Virol. 2019 May 26;91(9):1602–7. doi: 10.1002/jmv.25496 (PMC6772119; doi:10.1002/jmv.25496)
Supplement: Supplementary file 2 — Supporting information [file JMV-91-1602-s002.docx]

**Supplemental data**

**S1 Table. Characteristics of patients included and detection rates of the three swabs tested for the detection of HRSV by RT-qPCR**.
